# Potential phosphorylation of Liprin-α1 at threonine 701 regulates integrin-mediated cell motility

**DOI:** 10.1371/journal.pone.0337621

**Published:** 2025-12-01

**Authors:** Martina Ramella, Daniele Brambilla, Sara Surini, Diletta Tonoli, Lucrezia Maria Ribolla, Ivan de Curtis

**Affiliations:** 1 Vita-Salute San Raffaele University, Milan, Italy; 2 Cell Adhesion Unit, Division of Neuroscience, IRCCS San Raffaele Scientific Institute, Milan, Italy; University College Cork, IRELAND

## Abstract

A dynamic protein network at the leading edge of motile cells is needed to coordinate events required for efficient cell motility. Previous work has shown that the Ser/Thr kinase DYRK3 affects the assembly of this network, and phosphorylates its component Liprin-α1, a scaffold protein regulating adhesion turnover and cell motility. We have looked for phospho-sites of Liprin-α1 relevant for the regulation of cell motility, by examining the role played by serine/threonine residues phosphorylated within the intrinsically disordered regions of Liprin-α1. Phospho-null mutations within either the amino-terminal or the carboxy-terminal disordered regions affect Liprin-α1 phosphorylation induced by DYRK3. Functional analysis shows that mutations within the amino-terminal region do not affect cell motility, while a set of carboxy-terminal mutations reduces the positive effects of Liprin-α1 on cell spreading on the extracellular matrix. Among several candidate phospho-sites in this protein region, we identify Thr^701^ as one of the potential main targets of DYRK3 activity in Liprin-α1. The phospho-null mutation of Thr^701^ specifically inhibits Liprin-α1–induced potentiation of cell spreading on fibronectin. Our findings contribute to highlight the complexity of the regulation of Liprin-αprotein functions by phosphorylation/dephosphorylation events. Given the involvement of Liprin-α proteins in tumor cell motility and invasion, in-depth understanding of this regulatory complexity may highlight new possibilities for therapeutic intervention.

## Introduction

Specific regions of the cell membrane are enriched with scaffold proteins that act as platforms for dynamic supramolecular networks, playing a crucial role in regulating cellular processes like neurotransmitter release at synapses [1], tumor cell motility [2], and invadosomes [3]. Among these specialized regions are plasma membrane-associated platforms (PMAPs) [4], extended protein networks composed of scaffold proteins, including Liprin-α1, ERC1, and LL5s [5–7]. These structures support the extension of the front of migrating cells by regulating the turnover of adhesions [8–9]. We have recently shown that the Ser/Thr kinase DYRK3, a cytoplasmic member of the class II dual-specificity tyrosine-regulated kinases (DYRKs), is expressed in invasive breast cancer cells and regulates their migration and Matrigel invasion.

The term dual-specificity kinase is referred to the fact that DYRK kinases catalyze self-phosphorylation in the activation loop tyrosine. This event is mediated by a transitional intermediate version of DYRK resulting in a full kinase activation with a restricted kinase activity to Thr and Ser residues [10].

DYRKs belong to an evolutionarily conserved family of protein kinases that regulate cell differentiation, proliferation, and survival [10]. DYRK3 has been implicated in the regulation of multiple membrane-less organelles, also referred to as biomolecular condensates. Biomolecular condensates are dynamic molecular assemblies organizing the cytoplasm and nucleoplasm to perform specific cellular functions [11]. DYRK3 was shown to regulate a number of different condensates through reversible phosphorylation of intrinsically disordered regions (IDRs) of specific proteins, thus promoting the dissolution of P-granule-like stress granules [12], the disassembly of nuclear condensates like splicing speckles [13], and the clearance of proteins associated with the early secretory pathway [14]. Similarly, the nematode ortholog MBK-2/DYRK induces the disassembly of cytoplasmic P granules in *C. elegans* embryos [15].

We recently demonstrated that DYRK3 is essential for tumor cell motility: altered DYRK3 levels inhibit cell migration and Matrigel invasion [16]. Increased levels of DYRK3 activity inhibit PMAPs formation and the turnover of focal adhesions at the edge of migrating cells. Interestingly, we observed that increased DYRK3 activity specifically enhances the phosphorylation of the PMAP scaffold protein Liprin-α1, a promoter of tumor cell migration and focal adhesion turnover. The mammalian Liprin family includes four Liprin-α proteins and two Liprin-β proteins. Liprin-α proteins have an amino-terminal coiled-coil region required for dimerization [17] and for the interaction with different partners including KIF1A, ERC/ELKS proteins, and GIT family ARF-GAPs (ADP-ribosylation factor-GTPase-activating proteins) [18]. The carboxy-terminal portion of Liprin-α proteins includes three sterile alpha motifs (SAMs) responsible for the interaction with protein ligands including the Liprin-β proteins [5]. Moreover, Liprin-α proteins include predicted intrinsically disordered regions (IDRs), poorly structured flexible regions that have been implicated in the formation of supramolecular assemblies by liquid liquid phase separation [11]. The role of the phosphorylation of Liprin-α proteins by kinases including DYRK3 remains unclear, particularly in relation to the regulation of cell motility.

This study aims at clarifying the role of DYRK3-mediated phosphorylation of Liprin-α1 in regulating cell motility. We have screened for DYRK3-induced phosphorylation sites of Liprin-α1, and identified a specific Thr residue relevant for Liprin-α1-mediated regulation of cell motility.

## Materials and methods

### Plasmids

The following constructs including Liprin-α1 fragments have been produced and used in this study: FLAG-Liprin-N, FLAG-Liprin-N(S/A). The following full length phospho-mutants have been produced and used in this study: FLAG-fl-Liprin-α1-N(S/A); FLAG-fl-Liprin-α1-N(S/D); FLAG-fl-Liprin-α1-N1(S/A); FLAG-fl-Liprin-α1-N2(S/A); FLAG-fl-Liprin-α1-C(ST/A); FLAG-fl-Liprin-α1-C(ST/D); FLAG-fl-Liprin-α1-C1(ST/A); FLAG-fl-Liprin-α1-C1(ST/D); FLAG-fl-Liprin-α1-C2(ST/A); FLAG-fl-Liprin-α1-C2(ST/D); FLAG-Liprin-α1(T^701^A/S^708^A); FLAG-Liprin-α1(T^701^D/S^708^D); FLAG-Liprin-α1(S^741^A/S^745^A); FLAG-Liprin-α1(S^741^D/S^745^D); FLAG-Liprin-α1(T^701^A); FLAG-Liprin-α1(T^701^D); FLAG-Liprin-α1(S^708^A); FLAG-Liprin-α1(S^708^D). The phosphorylation mutants of Liprin-α1 (see S1–S3 Figs and S6–S7 Figs) were obtained by substitution of synthetic human Liprin-α1 cDNA fragments (from GeneArt, ThermoFischer Scientific) carrying the selected mutations into the plasmid pFLAG-Liprin-α1.

The plasmids coding for full length Liprin-α1 carrying single or double amino acid substitutions were generated by site-directed mutagenesis from the plasmid coding for wild type FLAG-liprin-α1. Specifically, the primers for FLAG-Liprin-α1(T^701^A/S^708^A) were 5′-gg cgctccgccccacgaaggatccctcacgccccagctcggg-3’ and 5’-cccgagctggggcgtgagggatccttcgtgggg cg gagcgcc-3’; for FLAG-Liprin-α1(S^741^A/S^745^A) primers 5’- ccataaagtgtgaaaccccccccttccccccga gagccc-3’ and 5’- gggctctcggggcggaaggggggcggtttcacactttatgg −3’; for FLAG-Liprin-α1 (T^701^ D/S^708^D) primers 5’-ggcgctccgacccacgaaggatccctcacgacccagctcgg-3’ and 5’-ccgagctgg gtcgtga gggatccttcgtgggtcggagcgcc-3’; for FLAG-Liprin-α1(S^741^D/S^745^D) primers 5’-ccataaagttgaaacc gacccccttccgacccagagccc-3’ and 5’-gggctctcgggtcgg aagggggtcggtttcacactttatgg-3’; for FLAG-Liprin-α1(T^701^A) primers: 5′-gggcgctccgctccacgaaggatc-3′ and 5′-gatccttcgtggagcggagcgccc-3; for FLAG-Liprin-α1(T^701^D) primers 5’-gggcgctccgatccacgaaggatc-3’and 5’-gatccttcgtggatcggagcgccc-3’; for FLAG-Liprin-α1(S^708^A) primers 5’-cgaaggatccctcacg ccccagctcggg-3’ and 5’-cccgagctggggcgtgagggatccttcg-3’; for FLAG-Liprin-α1(S^708^D) primers 5’-cgaaggatccctcacgacccagctcggg-3’ and 5’-cccgagctgggtcgtgagggatccttcg-3’.

### Antibodies

The antibodies used in this study include: anti-Liprin-α1 pAb (Proteintech); anti-ERC1 pAb (Sigma); anti-paxillin (clone 349; BD Biosciences); anti-DYRK3 pAb (R&D systems); mAbs anti-tubulin-α (Clone DM1A), anti-βActin pAb (Abcam), anti-Calnexin mAb (clone 37/ Calnexin; BD Transduction Laboratories) and pAbs anti-FLAG (Sigma); anti-GFP rabbit pAb (Thermo Scientific) and chicken pAb (Abcam). Alexa Fluor 647 Phalloidin (Thermo Scientific. Alexa-Fluor–conjugated secondary antibodies (Thermo Scientific).

### Cell line and transient transfection

COS7 cells were cultured in DMEM containing 10% fetal clone III (Hyclone), 100 U/ml penicillin, 100 μg/ml streptomycin, and 20 mM glutamine. For transient transfection, cells were seeded on plastic and transfected 24 or 48 h later with Lipofectamine-2000® (Thermo Fisher Scientific, Paisley, UK). The transfection mixtures included plasmid DNA in a range of 1–5 μg. Transfection was performed in Optimem medium, which was replaced with fresh growth medium after 4 h. The small molecule DYRK3 inhibitor GSK-626616 was diluted in serum- free medium (PubChem CID: 15981157, Tocris Bioscience) and added to cells washed in serum-free medium, following a described protocol [12].

### Cell Lysis, Immunoprecipitation, and Immunoblotting

Cells were placed on ice and washed twice with ice-cold TBS (150 mM NaCl, 20 mM Tris-HCl, pH 7.5) before lysis in 50–150 μl of lysis buffer containing 0.5% Triton X-100, 150 mM NaCl, 20 mM Tris-Cl pH 7.5, 1 mM sodium orthovanadate, 10 mM sodium fluoride, Complete™ protease inhibitor cocktail (Roche), 0.5 mM phenylmethylsulfonyl fluoride (Sigma-Aldrich), and 1 mM dithiothreitol. Lysis was carried out for 15 minutes at 4°C with continuous rotation. Insoluble material was removed by centrifugation at 16,000 × g for 10 minutes at 4°C. Protein concentration in the resulting supernatant was determined using the Bradford protein assay (Bio-Rad). For immunoprecipitation, cell lysates were incubated with GFP-Trap (Chromotek) before further processing for SDS-PAGE and immunoblotting. For immunoblotting, denatured lysates and immunoprecipitates were separated by SDS-PAGE and transferred onto 0.45 μm PROTRAN® nitrocellulose membranes (GE Healthcare Amersham Biosciences). Membranes were blocked with 5% (w/v) milk in TBST, followed by incubation with primary antibodies and HRP-conjugated secondary antibodies. Detection was performed using the Clarity ECL substrate and imaged with the ChemiDoc MP Imaging System (Bio-Rad). Protein quantification was performed using ImageLab software (Bio-Rad). For Liprin-α1 phosphorylation levels, the intensity of the band corresponding to the phosphorylated form (upper band) was quantified and divided by the total liprin intensity (sum of the upper and lower bands). The resulting value, expressed as a percentage, was subsequently normalized to GFP-DYRK3 expression levels in order to correct for possible differences in expression among samples. The values are shown in the graph, where each point represents an individual sample; the mean and standard error (SEM) are also indicated for each experimental group. This approach preserves the variance of the original data, allowing for an appropriate statistical analysis among groups.

**Fig 1:**
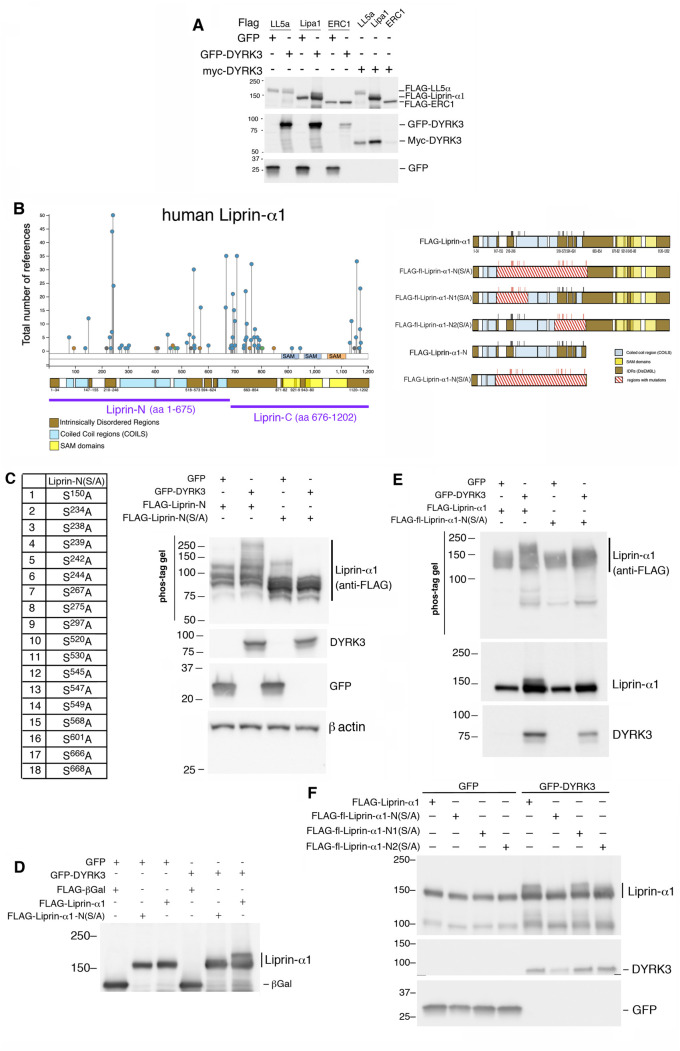
DYRK3 phosphorylates the amino-terminal IDRs of Liprin-α1. **(A)** Immunoblot from SDS-PAGE loaded with lysates (30 µg protein/lane) from COS7 cells co-transfected for 48 h with the indicated constructs. **(B)** Left: scheme of the phosphorylation sites (in blue) reported for human Liprin-α1 by PhosphoSitePlus (acetylation in green, ubiquitination in orange); below is shown the distribution of the predicted coiled-coils by the COILS program [19] and of the IDRs by the DisEMBL program [20] in the Liprin-α1 polypeptide. Right: scheme of the phosphomutants used in the experiments shown in panels **C-F**. For details see S2 Fig. **(C)** Ser/Thr residues mutated to Ala in the amino-terminal fragment Liprin-N(S/A). Right: COS7 cell co-transfected with GFP or GFP-DYRK3 with the indicated FLAG-tagged fragments. Immunoblotting from PhosTag gel (25 µM), 10 µg protein lysate/lane; 50 μg of protein lysate/lane were loaded on acrylamide gel to detect GFP-DYRK3 and GFP. **(D)** Immunoblotting on COS7 cell lysates (30 µg/lane) after transfection (48 h) with the indicated Liprin-α1 constructs. **(E)** COS7 cells lysed 48 h after transfection with full length Liprin-α1 proteins; 10 µg of protein lysate/lane (PhosTag gel). **(F)** COS7 cells transfected with the indicated full length Liprin-α1 proteins, lysed 48 h after transfection; 20 µg of protein lysate/lane.

**Fig 2:**
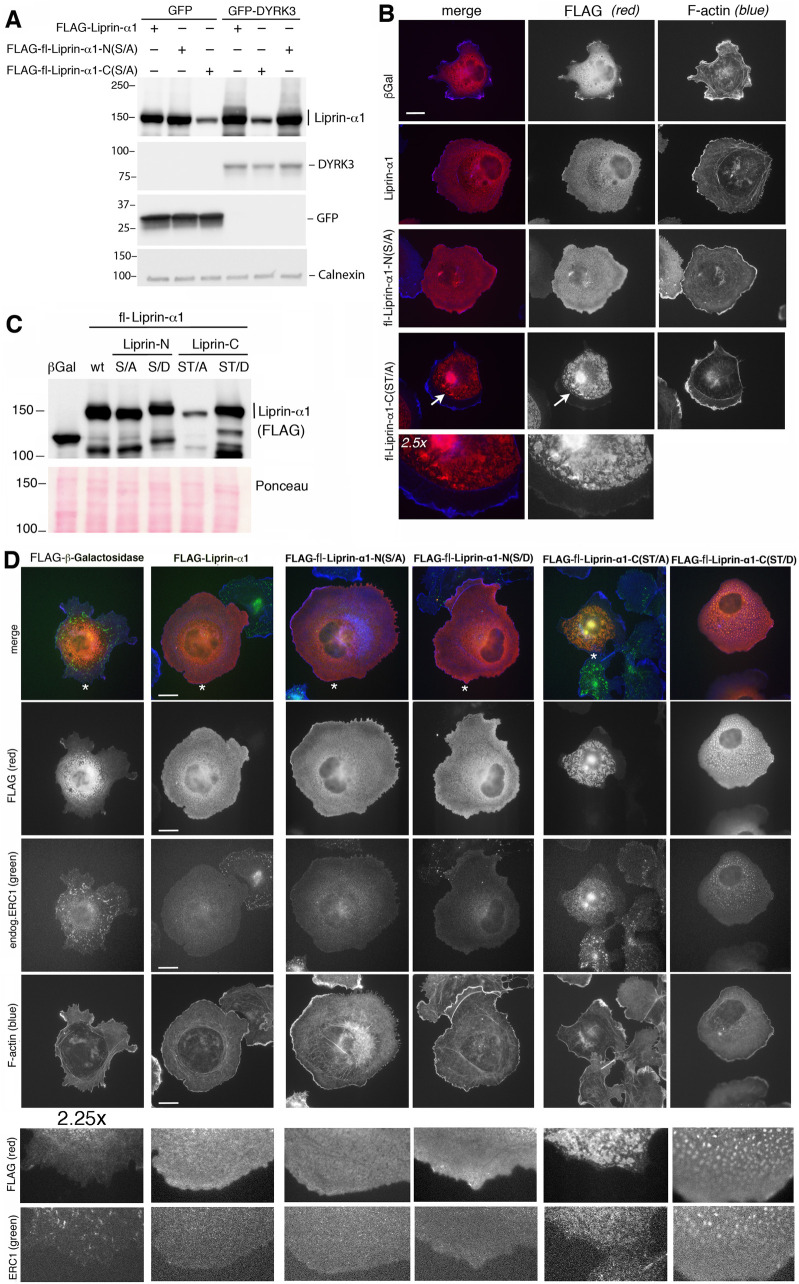
DYRK3 phosphorylates the carboxy-terminal IDRs of Liprin-α1. (**A**) Left: scheme of the phosphomutants used in the experiments shown in panels **A** and **C**. For details see S2, S3, S6, S7 Figs. Right: immunoblot from SDS-PAGE loaded with lysates (30 µg protein/lane) from COS7 cells co-transfected with the indicated constructs. (**B**) Immunofluorescence on COS7 cells transfected to express the indicated FLAG-tagged proteins (red) and fixed 1 h after replating on fibronectin. Co-staining with fluorescent phalloidin. Bar, 20 µm. Bottom images: enlargements (2.5x) of areas indicated with arrows in the corresponding lower magnifications. (**C**) COS7 cells transfected (24 h) with the indicated FLAG-tagged constructs. Below: Ponceau staining showing same amounts of total protein loaded in each lane (30 µg of protein lysate/lane). (**D**) COS7 cells transfected with the indicated FLAG-tagged constructs were replated for 1 h on fibronectin before fixation and immunostaining with anti-FLAG and anti-ERC1 antibodies to detect endogenous ERC1. Bars, 20 µm. Enlargements (2.25x) are shown of areas indicated by white asterisks in the top row images.

**Fig 3:**
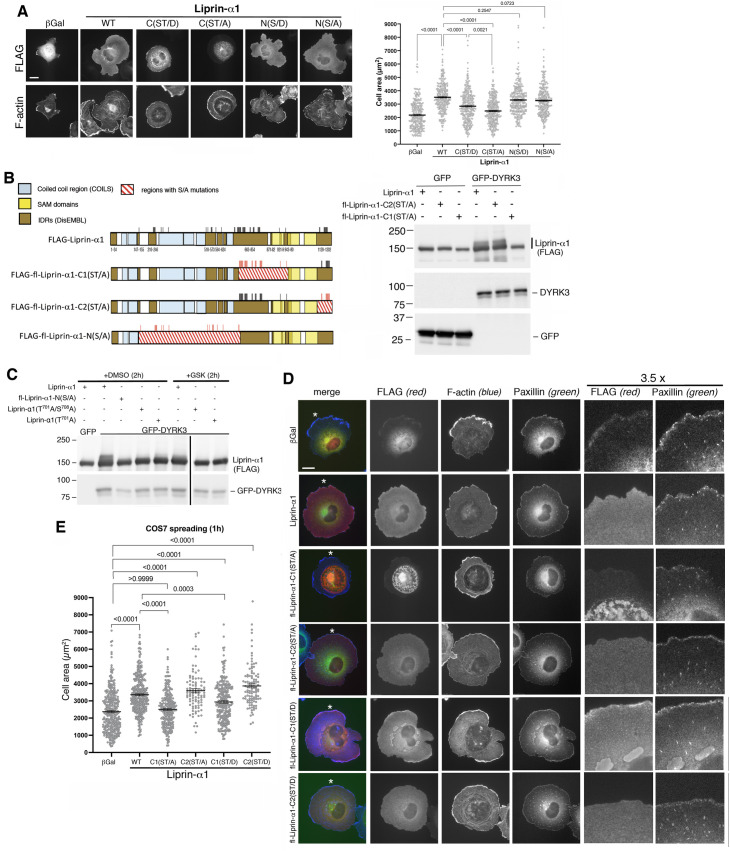
Phospho-mutants in the IDR Liprin-α1-C1 influence Liprin-α1–enhanced cell spreading. (**A**) Transfected COS7 cells replated for 1h on fibronectin before immunofluorescence. Bar, 20 µm. Right, quantification of the projected cell area (n = 260–288 cells from 3 independent experiments. Statistical analysis was performed using one-way ANOVA, Kruskal-Wallis test with Dunn’s posthoc). (**B**) Left: scheme of the phosphomutants carrying several mutations, used in the experiments shown in panels **B** and **C**. For details see S2, S3 Figs. Right: equal amounts of lysates (25 µg/lane) from COS7 cells co-transfected (48 h) with the indicated FLAG-tagged Liprin-α1 constructs together with either GFP or GFP-DYRK3. (**C**) COS7 cells co-transfected for 48 h with the indicated FLAG-tagged Liprin-α1 constructs together with either GFP or GFP-DYRK3 were incubated for 2 h with 1 µM of DYRK3 inhibitor (GSK-626616 small molecule) or with DMSO. Immunoblotting with 20 µg of protein lysate/lane. (**D**) Immunofluorescence of COS7 cells transfected with the indicated FLAG-tagged constructs. Immunostaining for FLAG (red), endogenous paxillin (green) and F-actin (blue). Bar, 20 µm. The last two panels on the right show enlargements (3.5x) of areas indicated by white asterisks in the left row images. (**E**) Quantification of the projected cell area of transfected COS7 cells plated for 1 h on fibronectin (n = 324–241 from 3 independent experiments; 96–104 cells from one experiment for fl-FLAG-Liprin-C2 mutants. Statistical analysis was performed using one-way ANOVA, Kruskal-Wallis test with Dunn’s posthoc).

**Fig 4:**
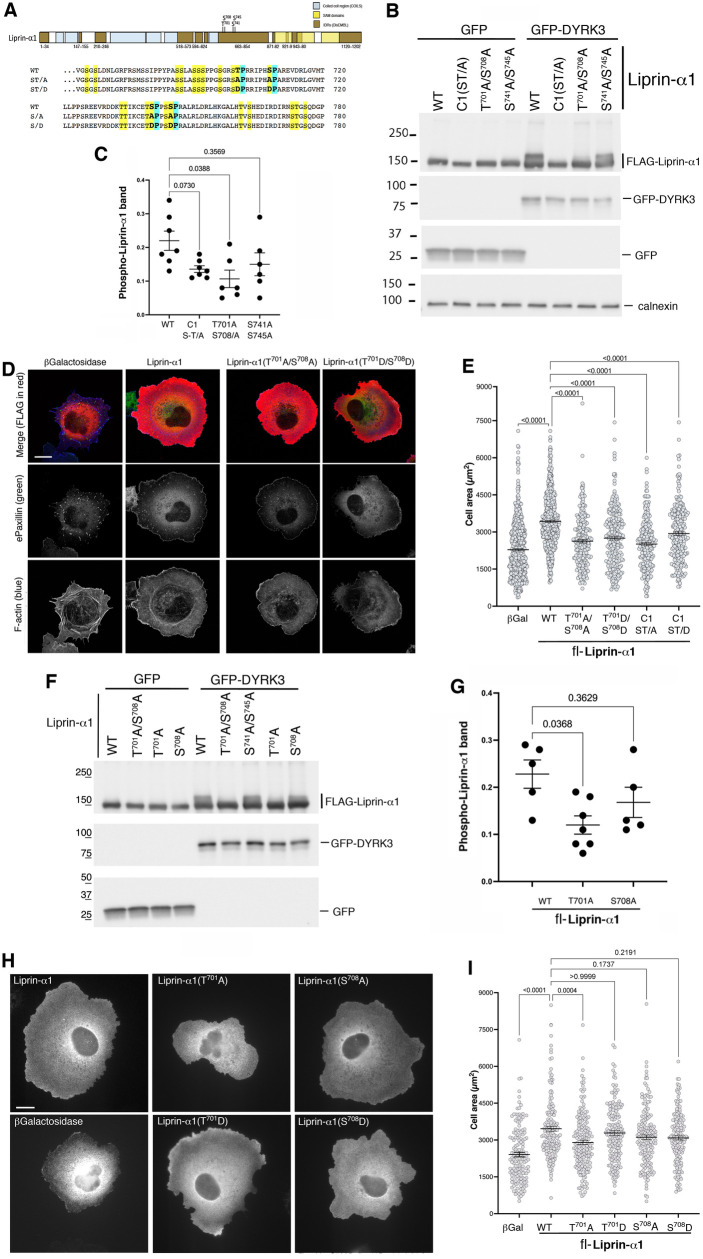
Identification of Thr^701^ as a DYRK3–induced phosphorylation site required to enhance cell motility. **(A)** Scheme and sequence showing the position of the four Ser and Thr residues (in bold) mutated within the IDR in the carboxy-terminal portion of Liprin-α1. **(B)** Immunoblot on lysates (20 µg protein/lane) from COS7 cells co-transfected with either GFP or GFP-DYRK3 together with the indicated FLAG-tagged full length Liprin-α1 constructs. **(C)** Quantification of the effect of the double mutations on the phosphorylation of Liprin-α1 (n = 6-7 samples; Brown-Forsythe and Welch ANOVA tests). **(D)** Morphology of COS7 cells transfected with the indicated full length FLAG-tagged constructs and immuno-stained 1 h after replating on fibronectin. FLAG (red), Paxillin (green), F-actin (blue). Bar, 20 µm. **(E)** Quantification of the projected cell area of transfected COS7 cells after spreading for 1 h on fibronectin (n = 229–584 cells, from 3–7 independent experiments. Statistical analysis was performed using one-way ANOVA, Kruskal–Wallis test with Dunn’s posthoc. The graph shows the means ±SEM). **(F)** Immunoblot on lysates (20 µg of protein/lane) from COS7 cells co-transfected with either GFP or GFP-DYRK3 together with the indicated full length FLAG-tagged Liprin-α1 constructs. **(G)** Quantification of the effect of the single amino acid substitutions on the phosphorylation of Liprin-α1 (n = 5-7 samples; Brown-Forsythe and Welch ANOVA tests). **(H)** Morphology of COS7 cells transfected with the indicated full length FLAG-tagged constructs and immuno-stained 1 h after replating on fibronectin. Bar, 20 µm. **(I)** Quantification of cell spreading on transfected COS7 cells were replated for 1h on fibronectin before immunostaining (as in **H**). The graph shows means ±SEM (n = 149-172 cells from 3 independent experiments. Statistical analysis was performed using one-way ANOVA, Kruskal–Wallis test with Dunn’s posthoc. The graph shows the means ±SEM).

Uncropped, unadjusted images of blots reported in Figs.1-4 are presented in **S1 Raw images**.

### Phos-tag gels

Transfected cells were lysed ans insoluble material removed by 15 min centrifugation at 16,000 RCF at 4°C. Untreated and phosphatase-treated cleared lysates (10 μg/lane) were denatured for 10 min at 70°C in 2 × LDS sample buffer (Invitrogen), and separated on 6–7% acrylamide gels with 25–50 μM PhosTag (Wako Chemicals, Japan), 100 μM Zn(NO_3_)_2_ in running buffer (50 mM MOPS, 50 mM Tris base, 0.1% SDS, 5 mM sodium bisulfite, pH 7.8). Gels were washed twice for 30 min each in transfer buffer with 1 mM EDTA (1 × NuPAGE Transfer Buffer, Invitrogen) for transfer overnight at 4°C to nitrocellulose membranes (Amersham) in 1 × NuPAGE Transfer Buffer with 10% methanol, 5 mM sodium bisulfite. Membranes were then processed for immunoblotting.

### Immunofluorescence

Cells were fixed for 10 min at room temperature using 3% paraformaldehyde, then permeabilized with 0.1% Triton X-100 in phosphate buffer saline. Samples were incubated with primary antibodies, washed, and subsequently incubated with appropriate secondary antibodies. Mounting was performed with ProLong Gold antifade reagent (Thermo Fisher Scientific). Imaging was carried out with Leica TCS SP5 and TCS SP8 SMD FLIM laser scanning confocal microscopes with an HC PL APO CS2 63 × objective (NA 1.4)

### Cell Spreading assay

COS7 cells were transfected with the indicated plasmid. After 24 h, 6x10⁴ cells were replated onto glass coverslips coated with human fibronectin (10 μg/ml, Corning). Cells were fixed 1 h after replating and processed for immunofluorescence. Images were captured using a Zeiss Axio Observer.Z1 inverted microscope with a 63x Plan-Apochromat lens (NA 1.4) and Hamamatsu 9100−02 EM CCD Camera. The projected cell area was quantified using ImageJ software.

### Statistical analysis

Statistical analysis was conducted using GraphPad Prism 9.0. Normality of all datasets was assessed with the Shapiro-Wilk test. For normally distributed datasets, statistical significance was determined using an unpaired two-tailed Student’s t test or one-way ANOVA with Dunnett’s or Tukey’s post-hoc analysis. For non-normally distributed datasets, the Kruskal-Wallis test with Dunn’s post-hoc analysis was applied. Data are expressed as mean ± SEM.

## Results and discussion

### DYRK3 induces the phosphorylation of IDRs in the amino-terminal region of Liprin-α1

Given the crucial role of Liprin-α1 in cell motility, and our finding that this PMAP component can be phosphorylated by DYRK3 [16], we have set to identify phosphorylation sites of Liprin-α1 that are relevant for the regulation of the function of this protein in cell motility. We confirmed that Liprin-α1 co-transfected in COS7 cells was specifically phosphorylated by either GFP-DYRK3 or Myc-DYRK3. Phosphorylation was detected by immunoblotting as the clear shift of a fraction of the protein to higher molecular weights. Some phosphorylation was observed also for LL5-α, but not for ERC1/ELKS (Fig 1A). In this study further analysis was performed on Liprin-α1.

IDRs are suitable sites of post-translational regulation including phosphorylation, due to their flexibility that facilitates access to enzymatic activities by regulatory enzymes including kinases and phosphatases [21]. DYRK3 is known to phosphorylate Ser and Thr residues in the IDRs of proteins involved in the formation of biomolecular condensates [14,15]. Phosphorylation within IDRs can modulate protein interactions and functions [22]. Analysis using the PhosphoSitePlus database (https://www.phosphosite.org/) [23] revealed the presence of several Ser and Thr residues concentrated in the predicted IDRs of Liprin-α1 (Fig 1B; UniProtKB Q13136). We focused on potential phosphorylation sites within the IDRs of Liprin-α1 that could be potential targets of the DYRK3 kinase. Specific phosphorylation sites in substrates of DYRK kinases may include a proline residue at position +1 and an arginine at position –3 [24]. This consensus for phosphorylation has been quite well defined for DYRK1A. On the other hand, the limited data available suggest a wide variation in the phosphorylation motifs found in the substrates of other family members including DYRK3 [10,25,26]. For this reason, we mutated several serine phosphorylation sites in the N-terminal region of Liprin-α1 identified in PhosphoSitePlus. Based on this analysis, we selected 18 serine residues to be mutated within the amino-terminal fragment Liprin-N (residues 1–675), since we recently found that DYRK3 induced a detectable phosphorylation of this region of Liprin-α1 [16]. To identify specific phosphorylation sites within this region, we generated the phospho-null mutant FLAG-Liprin-N(S/A) including 18 Ser to Ala residue substitutions (S/A) mostly within the amino-terminal IDRs of the Liprin-N region (Fig 1B) to prevent potential phosphorylation of any of these sites by the DYRK3 kinase (S1 Fig and S2 Fig). Analysis by immunoblotting on PhosTag gels showed that the 18 Ser/Ala phospho-null substitutions introduced into the N-terminal FLAG-Liprin-N fragment abolished the shift of the FLAG-Liprin-N(S/A) mutant fragment in cells co-expression of GFP-DYRK3 (Fig 1C).

We introduced the same 18 Ser/Ala substitutions (Fig 1C) into the N-terminal region of the full length Liprin-α1 to produce the fl-Liprin-α1-N(S/A) mutant protein. The phosphorylation induced by GFP-DYRK3 was strongly reduced in the fl-Liprin-α1-N(S/A) mutant (Fig 1D). We confirmed by immunoblotting from PhosTag gels that GFP-DYRK3 induces a clear shift of the wildtype Liprin-α1 protein, and a less evident shift of the fl.FLAG-Liprin-α1-N(S/A) mutant (Fig 1E).

To restrict the region including the phosphorylation site(s) responsible for the shift of the Liprin-α1 band induced by DYRK3, we produced two full length Liprin-α1 mutants: the fl-Liprin-α1-N1(S/A) mutant including the first 9 N-terminal Ser/Ala substitutions (from S^150^A to S^297^A, S1 Fig); and the fl-Liprin-α1-N2(S/A) mutant including the other 9 Ser/Ala substitutions (from S^520^A to S^668^A, S1 Fig). While DYRK3–induced phosphorylation of fl-Liprin-α1-N1(S/A) was similar to phosphorylation of the wildtype protein, phosphorylation of fl-Liprin-α1-N2(S/A) by DYRK3 was evidently reduced (Fig 1F). Therefore the mutation of the 9 Ser residues in fl-Liprin-α1-N2(S/A) affected more evidently the phosphorylation of Liprin-α1 by DYRK3. This result indicates that the Ser residues mutated in fl-Liprin-α1-N2(S/A) include one or more phosphorylation substrates for DYRK3. Of note, a partial smear of the Liprin-α1 band is detectable in the lysate from cells co-transfected with fl-Liprin-α1-N(S/A) and DYRK3 compared to the band in the control lysate from cells co-transfected with fl-Liprin-α1-N(S/A) and GFP (Fig 1E). This finding suggests the presence of additional sites of DYRK3-dependent phosphorylation outside the mutated N-terminal region, possibly in the carboxy-terminal IDRs of Liprin-α1 (Fig 1C).

### DYRK3 induces phosphorylation of IDRs in the carboxy-terminal region of Liprin-α1

To investigate the role of phosphorylation of the carboxy-terminal IDRs of Liprin-α1, we produced the full length protein fl-Liprin-α1-C(ST/A) carrying 36 phospho-null Ser/Ala (S/A) and Thr/Ala (T/A) substitutions (28 S/A and 8 T/A substitutions included in two regions with predicted IDRs within the carboxy-terminal part of human Liprin-α1 (Liprin-C in Fig 1B). Twenty six mutations (20 S/A and 6 T/A substitutions) were introduced into the IDR/C1 encompassing residues 663–980 of Liprin-α1; 10 mutations (8 S/A and 2 T/A substitutions) were introduced into the IDR/C2 encompassing residues 1120–1202 of Liprin-α1 (S1 Fig, S2 Fig, S3 Fig).

Biochemical analysis showed that the phospho-null mutant fl-Liprin-α1-C(ST/A) displayed a marked reduction in DYRK3-induced phosphorylation (Fig 2A). This mutant showed lower levels of the protein in the cell lysates compared to the wild type protein and to other mutants (S3 Fig). Immunofluorescence analysis of COS7 cells showed that cells expressing the fl-Liprin-α1-C(ST/A) mutant formed aggregate-like structures on the basal side of the cell as well as in the perinuclear region, when compared to either the wildtype Liprin-α1 or the fl-Liprin-α1-N(S/A) mutant (Fig 2B). This observation indicates that part of the mutant fl-Liprin-α1-C(ST/A) may be lost in the pellet after lysis due to the formation of aggregates, that may be induced by protein aggregation caused by the substitution of several polar Ser and Thr residues with nonpolar Ala residues.

To characterize the role played by the phospho-sites in the amino- and carboxy-terminal IDR regions of Liprin-α1, we produced the phospho-mimetic mutants (Ser and Thr residues changed to Asp) FLAG-fl-Liprin-α1-N(S/D) and FLAG-fl-Liprin-α1-C(ST/D) obtained by mutating the same residues changed to Ala in the FLAG-fl-Liprin-α1-N(S/A) construct (S1 Fig) and FLAG-fl-Liprin-α1-C(ST/A) construct (S2 Fig), respectively. Biochemical characterization by immunoblotting on lysates from transfected COS7 cells showed that both phospho-mimetic mutants FLAG-fl-Liprin-α1-N(S/D) and FLAG-fl-Liprin-α1-C(ST/D), as well as the phospho-null mutant FLAG-fl-Liprin-α1-N(S/A) were expressed at levels similar to the wildtype Liprin-α1 protein; in contrast to FLAG-fl-Liprin-α1-C(ST/A) that was strongly reduced compared to the other constructs (Fig 2C). Both phospho-mimetic mutants ran slightly more slowly on SDS-PAGE, as detected by the slightly increased molecular mass revealed by immunoblotting, probably due to the presence of several additional negatively charged Asp residues in these mutants compared to the wildtype protein.

Immunostaining was performed to look at effects and subcellular distribution of the mutants in cells spreading on fibronectin. Both phospho-null FLAG-fl-Liprin-α1-N(S/A) and phospho-mimetic FLAG-fl-Liprin-α1-N(S/D) showed a diffuse distribution and effects on the morphology of the cells similar to the one observed by cells overexpressing the wildtype Liprin-α1 [27]: cells appeared more spread than control cells (Fig 2D).

Distinct results were seen by mutants of phospho-sites in the carboxy-terminal IDRs. In contrast to the clustering/aggregation of FLAG-fl-Liprin-α1-C(ST/A) in COS7 cells, FLAG-fl-Liprin-α1-C(ST/D) was diffuse, with several cells displaying cytoplasmic round condensate-like formations of different sizes (Fig 2D), up to large condensates with irregular shapes (S4 Fig). These condensate-like structures suggest that they may originate by an increased tendency of phosphorylated Liprin-α1 to undergo liquid-liquid phase separation, as it has been recently demonstrated for the presynaptic family member Liprin-α3, that undergoes phase separation upon protein kinase C-dependent phosphorylation of Thr^760^, a residue that is not conserved in other Liprin-α family members [28].

In spreading COS7 cells, the endogenous scaffold protein ERC1/ELKS, a known PMAP component and a binding partner of Liprin-α1 [29] that promotes the turnover of focal adhesions [2], localizes near the edge of spreading/motile cells and near focal adhesions [16]. Increased expression of wildtype Liprin-α1 promotes cell spreading [27] and causes the relocalization of its binding partner ERC1/ELKS away from focal adhesions to become diffuse. As observed for wildtype Liprin-α1, also the expression of the fl-Liprin-α1-N(S/A) and fl-Liprin-α1-N(S/D) mutants induced the redistribution of endogenous ERC1 to become diffuse (Fig 2D). In cells expressing fl-Liprin-α1-C mutants, endogenous ERC1 colocalized at the clusters formed by fl-Liprin-α1-C(ST/A), and at the condensate-like structures formed by fl-Liprin-α1-C(ST/D), respectively (Fig 2D).

### The phosphorylation of IDRs in the carboxy-terminal region of Liprin-α1 influence Liprin-α1–enhanced cell spreading

The analysis of the effects of the phospho-mutants in different regions of Liprin-α1 provided us hints about the IDR(s) of Liprin-α1 that may be relevant for function. We analyzed the effects of the Liprin-α1 phospho-mutants on cell motility, by quantitatively addressing their effects on integrin-mediated cell spreading on the extracellular matrix protein fibronectin, that requires the dynamic rearrangement of both integrin-mediated focal adhesions and actin cytoskeleton [30]. Given the positive effects of Liprin-α1 overexpression on cell spreading and the inhibitory effects of endogenous Liprin-α1 depletion on spreading and motility [8,27], COS7 cell spreading represents a convenient and rapid motility assay to detect the effects of the Liprin-α1 phospho-mutants.

All four phospho-mutants induced a significant increase of the cell area compared to the control cells expressing βGalactosidase. In particular, the two amino-terminal mutants, phospho-null FLAG-fl-Liprin-α1-N(S/A) and phospho-mimetic FLAG-fl-Liprin-α1-N(S/D) increased cell spreading to an extent similar to that observed by cells expressing wildtype Liprin-α1 (Fig 3A). As previously shown for wildtype Liprin-α1, the increase in cell spreading induced by the fl-Liprin-α1-N(S/A) mutant was prevented by overexpression of DYRK3 (S5 Fig). This result is in agreement with our previous findings suggesting a competition between Liprin-α1–induced potentiation of cell spreading, and DYRK3-induced inhibition of cell spreading [16]. On the other hand the increase in cell spreading induced by either the phospho-null FLAG-fl-Liprin-α1-C(ST/A) or by the phospho-mimetic FLAG-fl-Liprin-α1-C(ST/D) mutant was significantly reduced compared to cells expressing wildtype Liprin-α1. These results indicate that only phospho-sites included in the carboxy-terminal IDRs of Liprin-α1 may play a role in regulating cell spreading.

To further restrict the region(s) relevant for the regulation of cell motility, we next produced phospho-null mutants fl-Liprin-α1-C1(ST/A) and fl-Liprin-α1-C2(ST/A) containing respectively 26 and 10 of the Ser/Thr to Ala substitutions included in the carboxyterminal IDRs (S6 Fig); in parallel we also produced phospho-mimetic mutants fl-Liprin-α1-C1(ST/D) and fl-Liprin-α1-C2(ST/D) containing the corresponding 26 and 10 Ser/Thr to Asp substitutions, respectively (S7 Fig). While DYRK3-induced phosphorylation of FLAG-fl-Liprin-α1-C2(ST/A) was similar to the phosphorylation of the wildtype protein, phosphorylation of FLAG-fl-Liprin-α1-C1(ST/A) by DYRK3 was strongly reduced, suggesting that the residues mutated in FLAG-fl-Liprin-C1(ST/A) include one or more phosphorylation sites for DYRK3 (Fig 3B). Residual phosphorylation was abolished by incubation of the transfected cells for 2 h with a DYRK3–specific inhibitor (Fig 3C). Morphological analysis by immunofluorescence showed that the FLAG-fl-Liprin-α1-C1(ST/A) mutant formed clusters/aggregates similar to those observed in cells expressing FLAG-fl-Liprin-α1-C(ST/A) (Fig 3D); while the FLAG-fl-Liprin-α1-C2(ST/A) mutant had a diffuse cytoplasmic distribution similar to the wildtype Liprin-α1, but induced also the formation of condensate-like structures as observed for the FLAG-fl-Liprin-α1-C(ST/D) mutant (Fig 2D; S4 Fig). The relocalization of endogenous ERC1/ELKS was still observed in cells expressing the phospho-mutants: while wildtype Liprin-α1 induced a diffuse distribution of endogenous ERC1, this protein colocalized with the central clusters formed by fl-Liprin-α1-C1(ST/A), and at the condensate-like structures formed by fl-Liprin-α1-C1(ST/D), respectively (S8 Fig). Quantification of the levels of expression of the different constructs in the transfected cells revealed that the presence of condensates in the C-terminal serine/threonine phosphomimetic mutants was not due to increased levels of the protein compared to the constructs not forming condensates (S9 Fig).

Quantification of COS7 cell spreading after 1 h on fibronectin showed that of the four phospho-mutants tested, only the phospho-null FLAG-fl-Liprin-α1-C1(ST/A) abrogated the stimulation of cell spreading. The other three constructs significantly increased cell spreading compared to control cells (βGal), although the phospho-mimetic FLAG-fl-Liprin-α1-C1(ST/D) mutant reduced significantly the increase in cell spreading compared to wildtype Liprin-α1. Both FLAG-fl-Liprin-α1-C2(ST/A) and and FLAG-fl-Liprin-α1-C2(ST/D) promoted spreading to the same extent of wildtype Liprin-α1 (Fig 3D). These results point to a specific role of the phosphorylation sites in the IDR of the Liprin-α1-C1 region in the regulation of cell motility.

### Phosphorylation of Liprin-α1 at Thr^701^ regulates cell spreading

Our biochemical, morphological and functional analysis with distinct sets of phopsho-mutants indicate that the IDRs within the Liprin-α1-C1 region (S7 Fig) may include phospho-site(s) relevant for the regulation of Liprin-α1 function. We next aimed at identifying the specific site(s) within this region by looking at Ser and Thr sites included in sequences compatible with a consensus sequence for DYRK3 [10,25,26]. We identified two pairs of residues followed by a Pro residue: Thr701/Ser708, and Ser741/Ser745. We generated phospho-null double mutants for each pair of residues within the full-length Liprin-α1 (Fig 4A). We tested the effects of the mutations on the phosphorylation of Liprin-α1 induced by DYRK3. Analysis by immunoblotting showed that co-expressing Liprin-α1(T^701^A/S^708^A) with DYRK3 resulted in a marked reduction of the phosphorylated form of the protein (detected as a decreased intensity of the higher molecular weight smear) compared to either the wildtype Liprin-α1 or the Liprin-α1(S^741^A/S^745^A) double mutant, that showed a less strong reduction of the higher molecular weight smear (Fig 4B). We checked whether the use of Phos-Tag gels would improve the resolution of the band shifted upon phosphorylation. We observed that contrary to the clear shifts observed by the smaller Liprin-α1 fragments (Fig 1D), the shift observed by Phos-Tag for the full length wildtype and mutants were less obvious (Fig 1E, S10 Fig), and insufficient to detect differences between wildtype and single mutations (S10 Fig). Therefore we performed the quantification on several replicates of blots from regular SDS-PAGE gels. Quantification of the signal of the higher molecular weight smear corresponding to phosphorylated Liprin-α1 (normalized to the signal of the whole Liprin-α1 band and to DYRK3 expression levels) confirmed that DYRK3 activity predominantly targeted the T701 and/or S708 sites, while no significant effect was detected on the phosphorylation of the Liprin-α1(S^741^A/S^745^A) phospho-null mutant (Fig 4C).

Contrary to the clustering/aggregation observed with the phospho-null mutant fl-Liprin-α1-C1(ST/A), the cytoplasmic subcellular distribution of the double mutant Liprin-α1(T^701^A/S^708^A) was diffuse, as for the wildtype protein (Fig 4D). Functionally, the increase in cell spreading induced by wildtype Liprin-α1 was significantly reduced by mutating the two residues T701 and S708 within the Liprin-α1-C1 region. Significant reduction of the projected cell area was observed by cells expressing either the phospho-null mutant FLAG-Liprin-α1(T^701^A/S^708^A), or the phospho-mimetic mutant FLAG-Liprin-α1(T^701^D/S^708^D) (Fig 4E, S11 Fig).

We tested the effects of single mutants Liprin-α1(T^701^A) and Liprin-α1(S^708^A) on DYRK3–induced phosphorylation and function. Quantitative analysis of band shift showed a clear and significant decrease of phosphorylation forLiprin-α1(T^701^A) compared to Liprin-α1(S^708^A) (Fig 4F-G). Analysis of the effects of single mutants on COS7 cell spreading revealed a significant decrease of Liprin-α1–stimulated spreading by the phospho-null Liprin-α1(T^701^A) mutant, but not by the Liprin-α1(S^708^A) mutant, nor by either one of the phospho-mimetic Liprin-α1(T^701^D) or Liprin-α1(S708D) variants. These findings highlight the specific role of Thr-701 phosphorylation in regulating cell motility.

## Conclusions

Dynamic protein networks at the leading edge of cells are needed to coordinate events required for efficient cell motility, including the reorganization of the cytoskeleton and the turnover of adhesions. The PMAP network includes scaffold proteins relevant for the regulation of these processes [4]. How this protein network is regulated to guide a dynamic process like cell motility needs to be determined. Recent studies indicate that Liprin-α proteins play a dual role in the formation of biomolecular condensates: both as structural components promoting ERC1-induced phase separation [31], but also as adaptors to recruit signaling enzymes at specific sites near the plasma membrane [32,33]. In this direction we have recently shown that the PP2A phosphatase and the DYRK3 kinase affect the formation of PMAPs with consequences on cell motility [16,34]. In the present study we have explored the possibility that phosphorylation of the IDRs of Liprin-α1 may be involved in the regulation of PMAPs dynamics, as proposed and shown for the phosphorylation of components of other macromolecular assemblies [22,35,36]. By exploring several possible phosphorylation sites within the IDRs of Liprin-α1, we have identified Thr^701^ as a substrate of the DYRK3 kinase and a potential important point of control of the Liprin-α1–mediated PMAP network, since mutation of Thr^701^ affects cell motility by reducing the promotion of cell spreading compared to the wild type Liprin-α1 protein.

Thr^701^ is conserved in all human Liprin-α1-α4 family members and in *Drosophila* Liprin-α, while the corresponding residue is a serine in *C. elegans* Liprin-α (S12 Fig) Other studies have highlighted the importance of the phosphorylation of Liprin-α proteins in distinct contexts. Interestingly, the phosphorylation of the same Thr^701^ residue of Liprin-α1 by the Cdk5 kinase has been shown to be critical for the maturation of excitatory synapses by regulation of the synaptic localization of PSD-95. In this context, phosphorylation of Thr^701^ decreases in response to neuronal activity, increasing the colocalization of Liprin-α1 with PSD-95, suggesting an inhibitory role of Liprin-α1 phosphorylation on the maturation of excitatory synapses [37]. Phosphorylation of the presynaptic family member Liprin-α3 at Ser^760^ by the protein kinase C promotes Liprin-α3 phase separation, leading to the formation of condensates that recruit RIM1α and Munc13−1, modulating the structure and function of the presynaptic active zone [28].

Interestingly, synthetic peptide libraries have been used to profile the substrate sequence specificity of hundreds of human Ser/Thr kinases [38]. This study reveals that T701 of Liprin-α1 may be a suitable substrate for both Cdk5 and DYRK3, but also for several other kinases from this and other kinase families. The analysis of the mechanisms by which Liprin-α1 is targeted by different kinases in different cellular contexts remains an important open question that deserves further investigation.

One intriguing aspect of the role of Liprin-α phosphorylation comes from the recent finding that the *C. elegans* presynaptic homolog Liprin-α/SYD-2 may undergo a conformational change between a closed and an open conformation, upon phosphorylation of three conserved Ser residues in the SAM-1 domain of SYD-2 by the neuronal SAD-1 kinase that promotes SYD-2/liprin-α phase separation and presynaptic active zone assembly [39]. On the other hand, the phosphatase PTP-3/LAR influences SYD-2/liprin-α activation by dephosphorylating Tyr^741^ of SYD-2/liprin-α. The non-phosphorylatable SYD-2-Y^741^F mutant is predominantly inactive, while the phospho-mimetic SYD-2-Y^741^E mutant is in an active conformation, supporting the model of activation/inactivation of Liprin-α/SYD-2 by phosphorylation/dephosphorylation, respectively. The closed conformation of liprin-α is expected to prevent interactions with other partners like ELKS/ERC1 and GIT1, important for the assembly of synaptic networks [40], while the phosphorylated open conformation allows Liprin-α to interact with its molecular partners [39]. We propose that the model suggested for the assembly of a presynaptic functional network may also apply to highly dynamic PMAPs forming at the front of motile cells. Our findings contribute to highlight the complexity of the regulation of Liprin-αprotein functions by phosphorylation/dephosphorylation events. Given the involvement of Liprin-α proteins in physiological and pathological processes like cancer [41], the in-depth understanding of this regulatory complexity may highlight new possibilities for therapeutic intervention.

## Supporting information

S1 Fig Alignment of sequences of wild type and phospho-mutant Liprin-α1 proteins. (TIF)

S2 Fig Scheme of wild type Liprin-α1 and amino-terminal S/A phospho-mutants. (TIF)

S3 Fig Scheme of wild type Liprin-α1 and carboxy-terminal S/A phospho-mutants. (TIF)

S4 Fig Subcellular distribution of FLAG-fl-Liprin-α1-(S/D) phospho-mutant. COS7 cell transfected with FLAG-fl-Liprin-α1-(S/D) and immunostained with anti-FLAG antibody and with phalloidin. Bar, 20 µm.
(TIF)

S5 Fig DYRK3 inhibits Liprin-α1–induced increased in cell spreading. Left: COS7 cells co-transfected with the indicated constructs were fixed 1 h after replating on fibronectin. FLAG (blue), GFP (green), F-actin (red). Bar, 20 µm. Right: quantification of the projected cell areas (n = 150–210 cells from 4 independent experiments; one-way ANOVA, Kruskal-Wallis test with Dunn’s posthoc). The graph shows the means ±SEM.
(TIF)

S6 Fig Scheme of wild type Liprin-α1 and amino-terminal S/D phospho-mutants. (TIF)

S7 Fig Scheme of wild type Liprin-α1 and carboxy-terminal S/D phospho-mutants. (TIF)

S8 Fig Morphology of COS7 cells expressing carboxy-terminal S/A or S/D mutants. COS7 cells transfected with the indicated FLAG-tagged constructs were fixed 1 h after replating on fibronectin. FLAG (red), endogenous ERC1 (green). Bar, 20 µm.
(TIF)

S9 Fig Expression levels for different Liprin-α1 proteins. For each transfection condition, COS7 cells were analyzed after 1 h of spreading on fibronectin and processing for immunofluorescence. In each cell, the average fluorescence intensity (arbitrary units) of the FLAG-tagged Liprin-α1 construct was measured, and the presence of protein condensates was assessed. Condensates were defined as discrete round structures in the cytoplasm. Positive cells contained at least 3 small condensates or at least a large condensate-like structure. In the graph, each point represents an individual cell (n = 35–50 from two experiments): black and yellow dots indicate cells with condensates and without condensates, respectively.
(TIF)

S10 Fig Phos-Tag of. Lysates (10 µg/lane) from COS7 cells transfected for 48 h with the indicated full length Liprin-α1 constructs were analyzed by immunoblotting from Phos-Tag gels (6% acrylamide, 25 µM Phos-Tag).
(TIF)

S11 Fig Morphology of COS7 cells expressing T^701^A/S^708^A or T^701^D/S^708^D mutants. COS7 cells transfected with the indicated FLAG-tagged constructs were fixed 1 h after re-plating on fibronectin, and immuno-stained with anti-FLAG antibody. Bar, 20 µm.
(TIF)

S12 Fig T^701^ is conserved among human and Drosophila Liprin-α’s. Sequence alignment of the region including residue T^701^ of human Liprin-α1: four human Liprin-α family members with *Drosophila* and *C. elegans* Liprin-α are shown.
(TIF)

S1 Raw images Uncropped, unadjusted images blots reported in Fig.1–4. (TIF)
