# Supplementary figures and images for: Potential phosphorylation of Liprin-α1 at threonine 701 regulates integrin-mediated cell motility
Source: PLoS One. 2025 Dec 1;20(12):e0337621. doi: 10.1371/journal.pone.0337621 (PMC12668518; doi:10.1371/journal.pone.0337621)

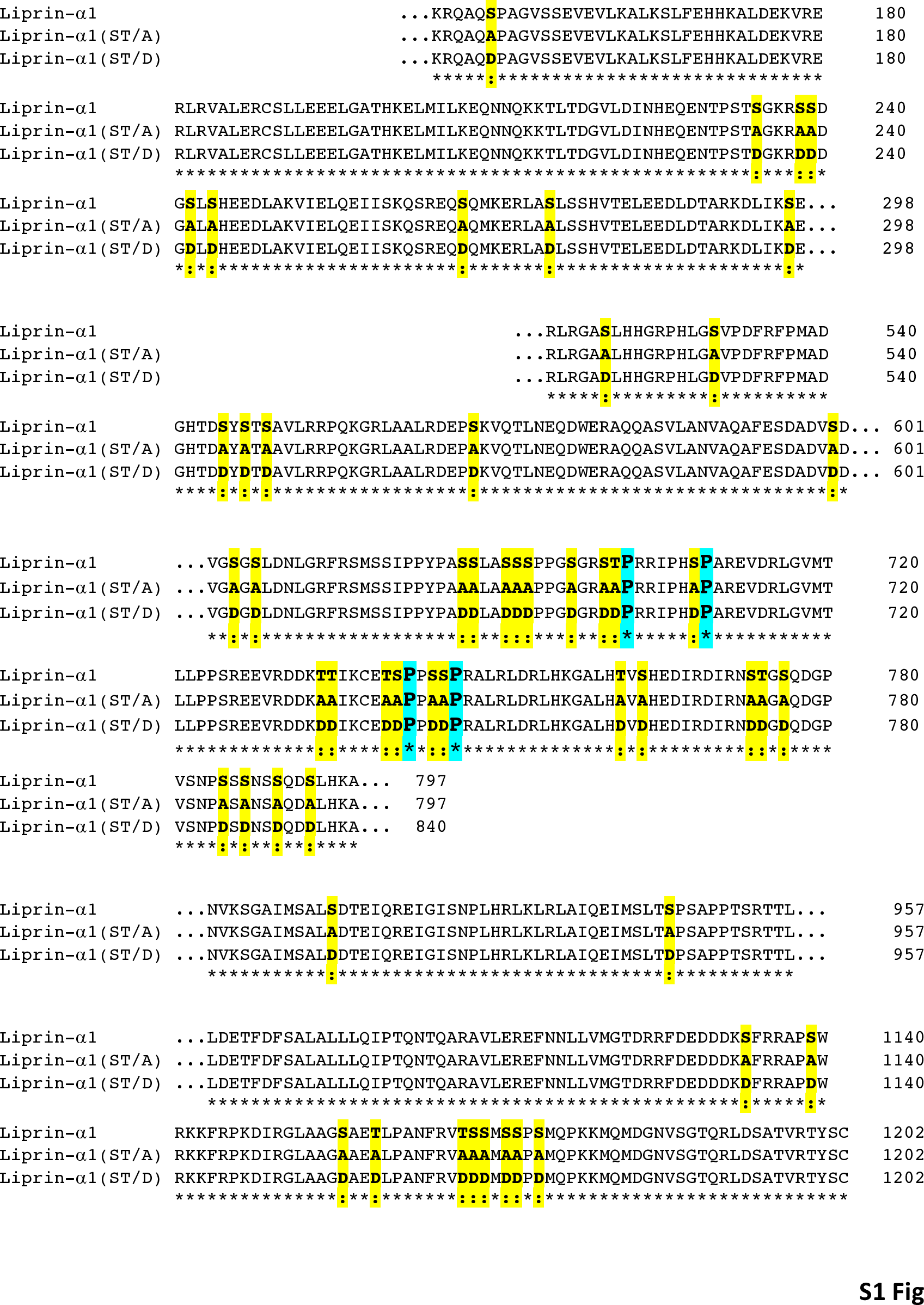

Supplement: S1 Fig — (TIF) [file pone.0337621.s001.tif]

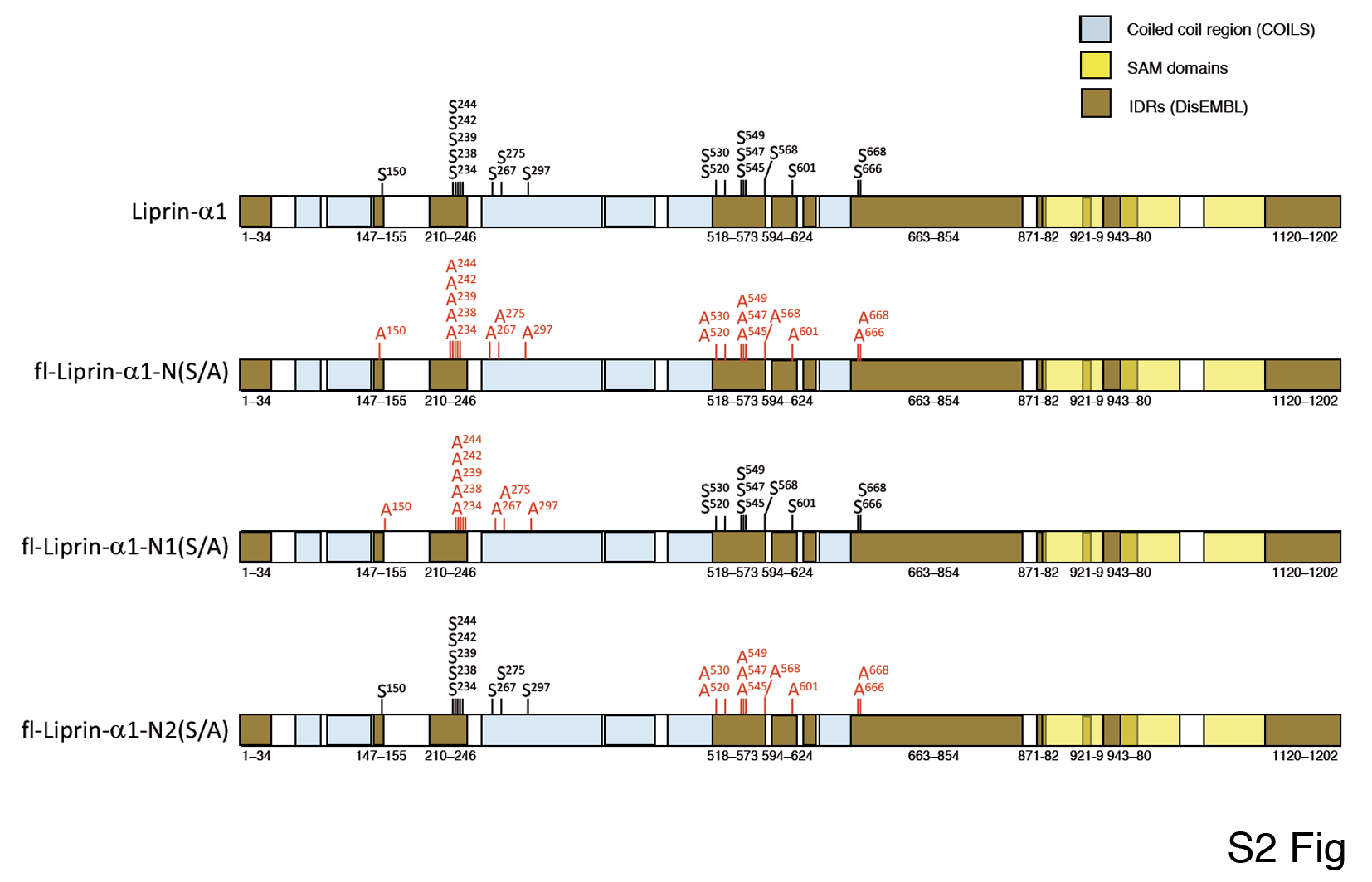

Supplement: S2 Fig — (TIF) [file pone.0337621.s002.tif]

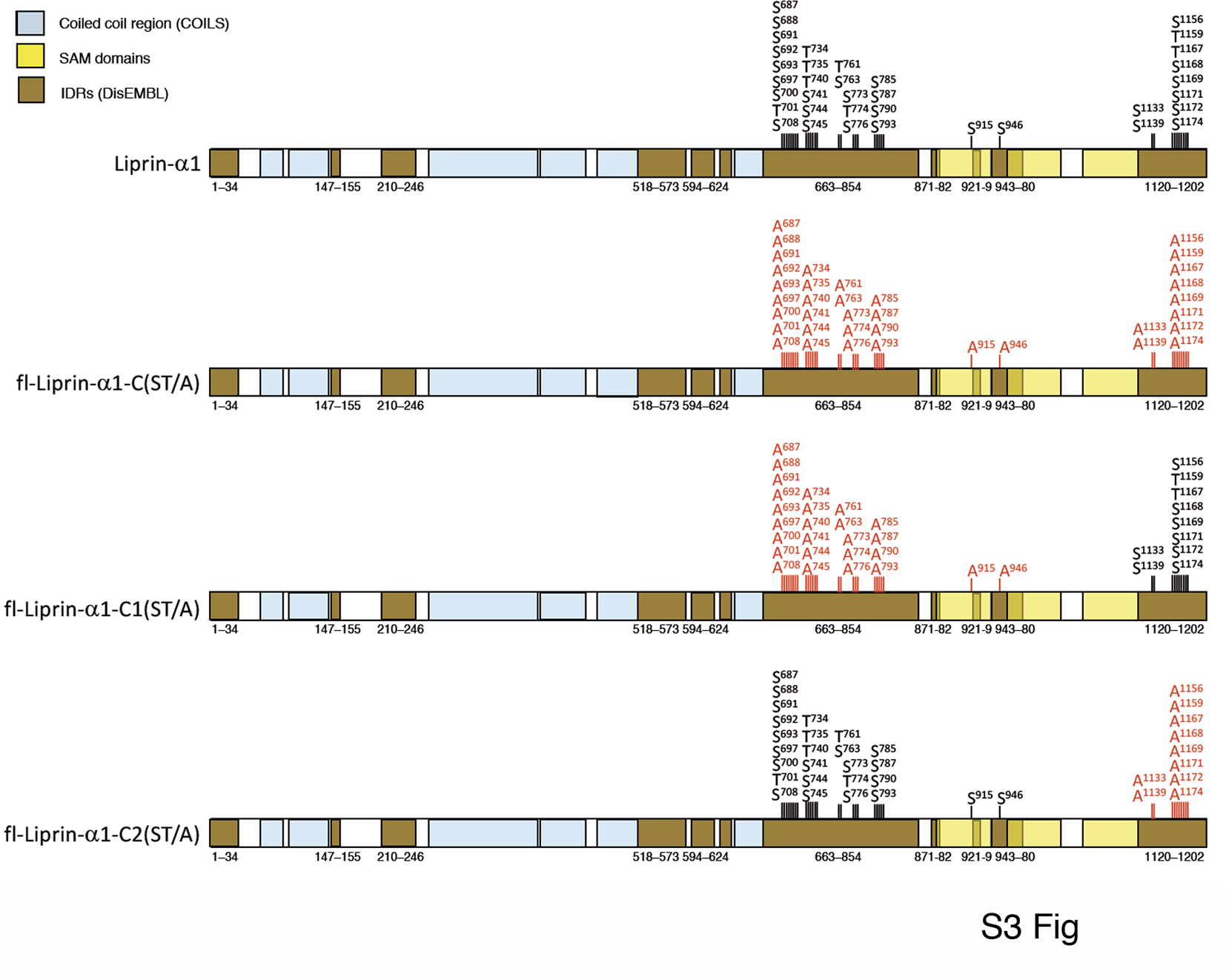

Supplement: S3 Fig — (TIF) [file pone.0337621.s003.tif]

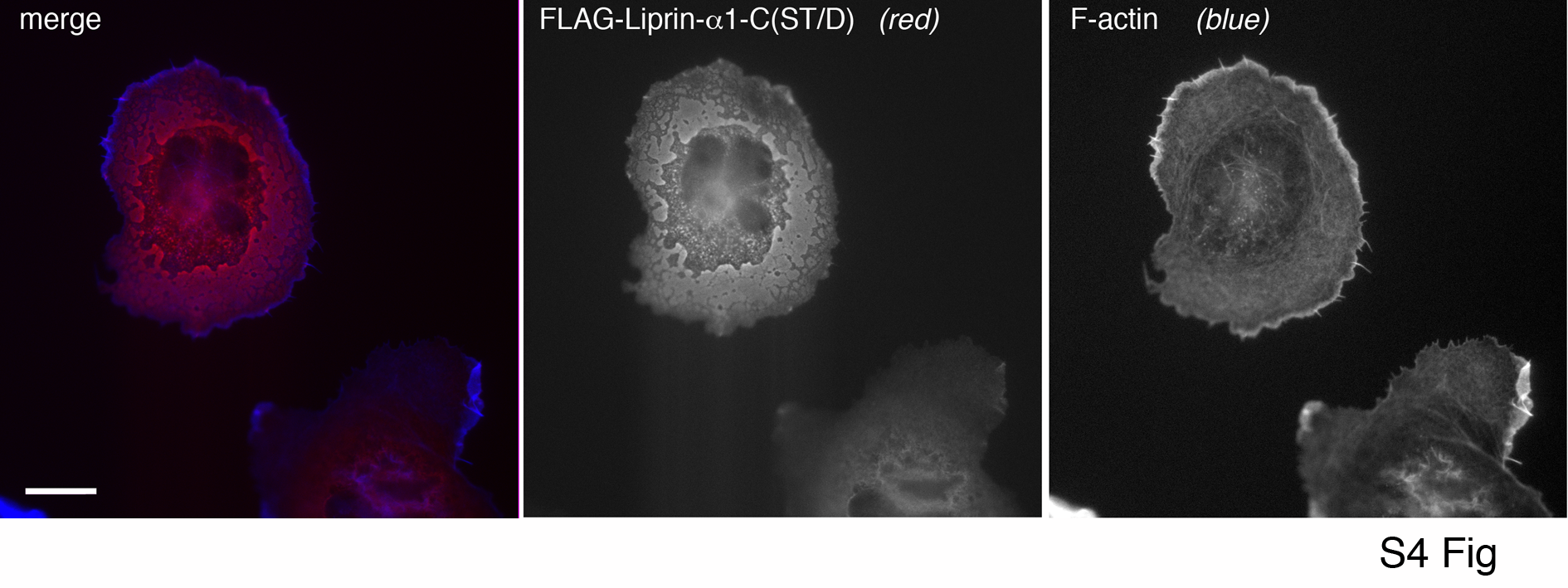

Supplement: S4 Fig — COS7 cell transfected with FLAG-fl-Liprin-α1-(S/D) and immunostained with anti-FLAG antibody and with phalloidin. Bar, 20 µm. (TIF) [file pone.0337621.s004.tif]

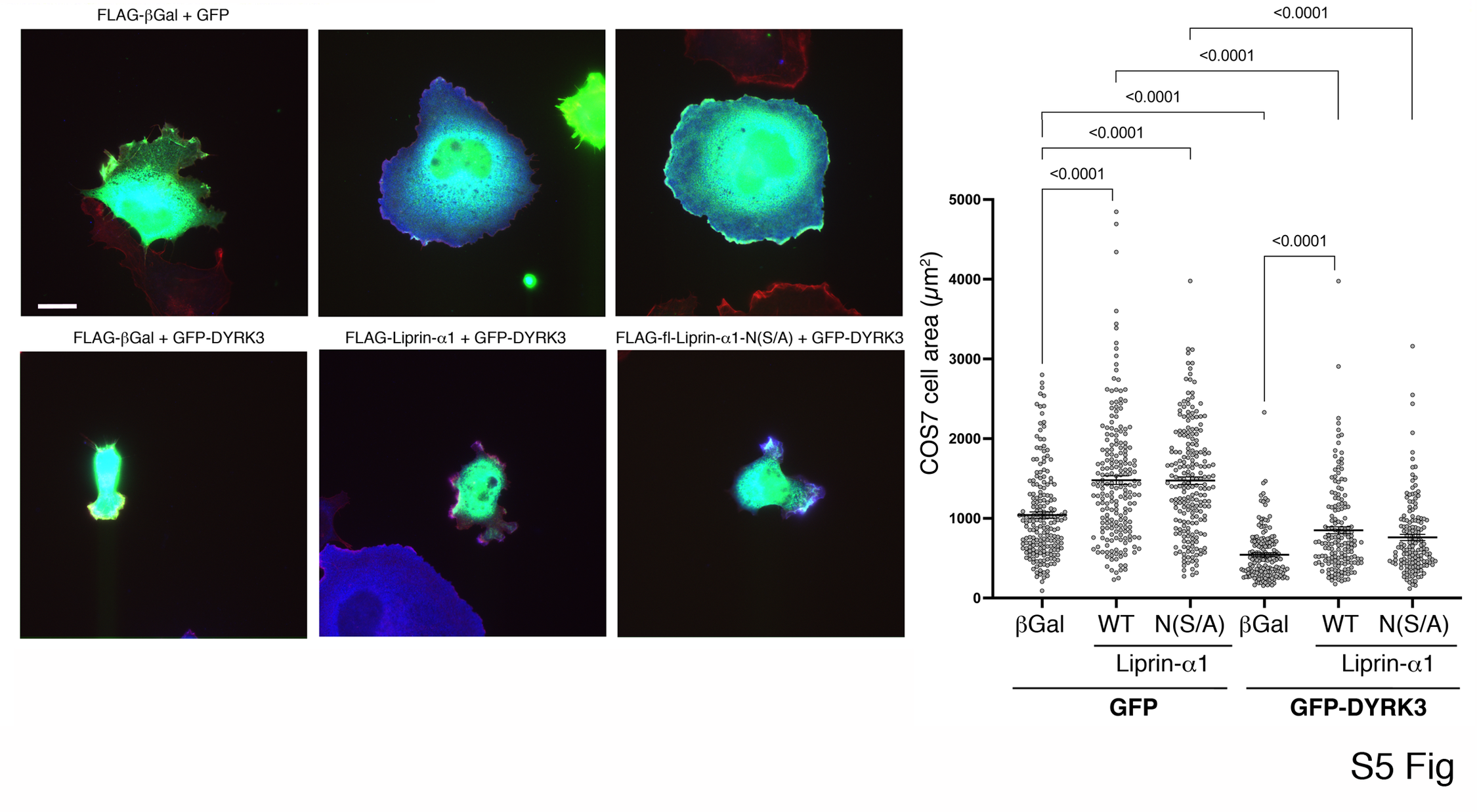

Supplement: S5 Fig — Left: COS7 cells co-transfected with the indicated constructs were fixed 1 h after replating on fibronectin. FLAG (blue), GFP (green), F-actin (red). Bar, 20 µm. Right: quantification of the projected cell areas (n = 150–210 cells from 4 independent experiments; one-way ANOVA, Kruskal-Wallis test with Dunn’s posthoc). The graph shows the means ±SEM. (TIF) [file pone.0337621.s005.tif]

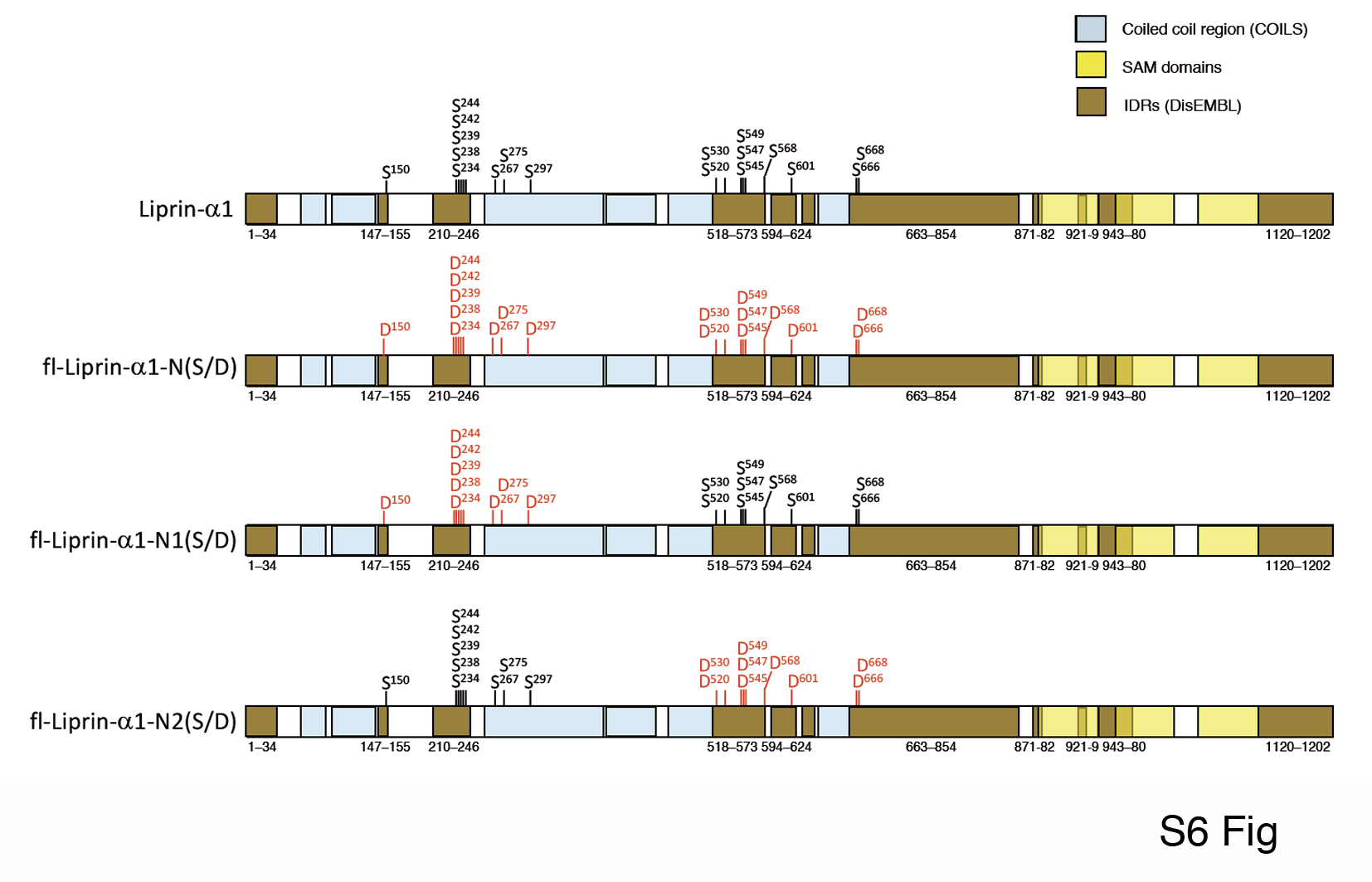

Supplement: S6 Fig — (TIF) [file pone.0337621.s006.tif]

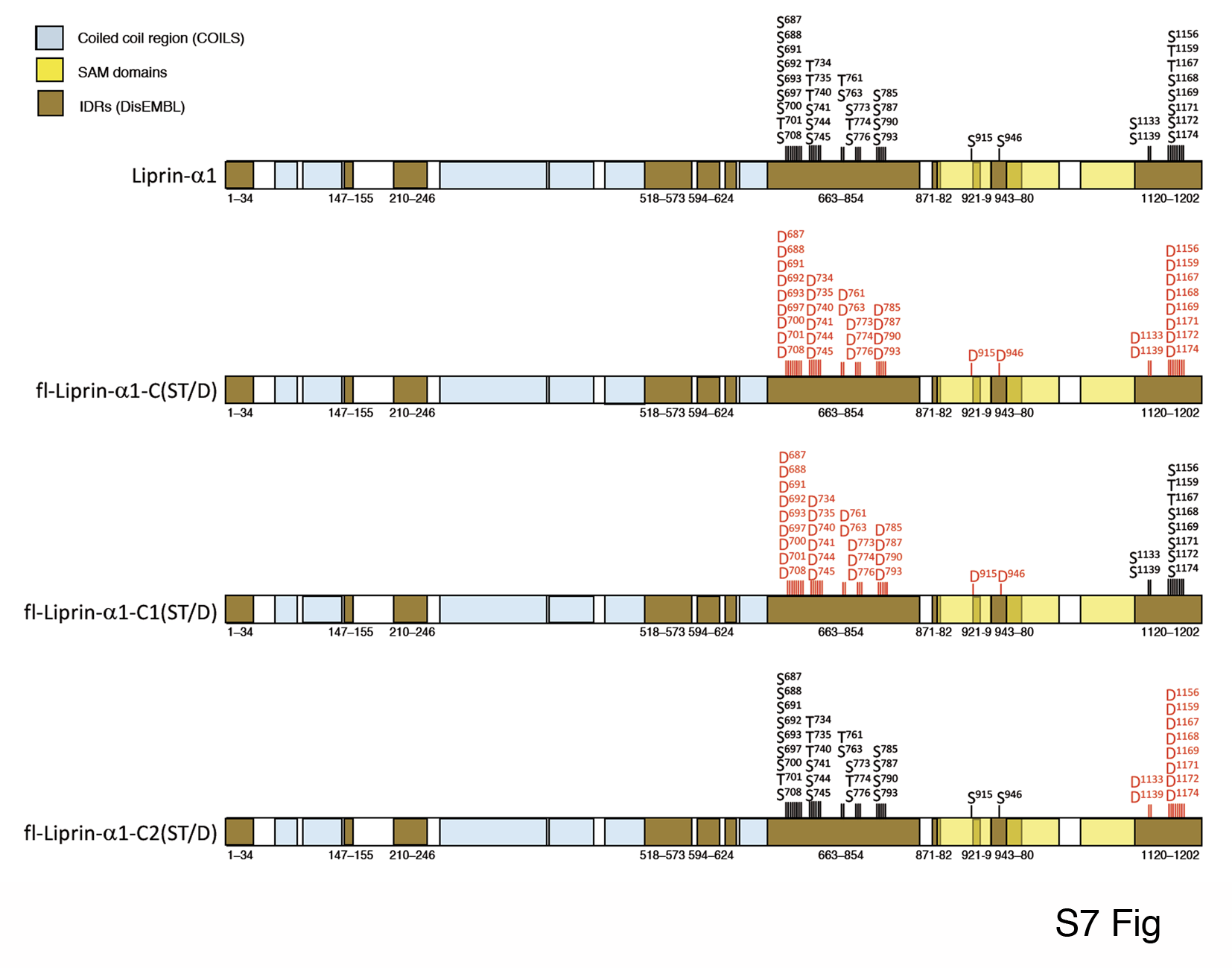

Supplement: S7 Fig — (TIF) [file pone.0337621.s007.tif]

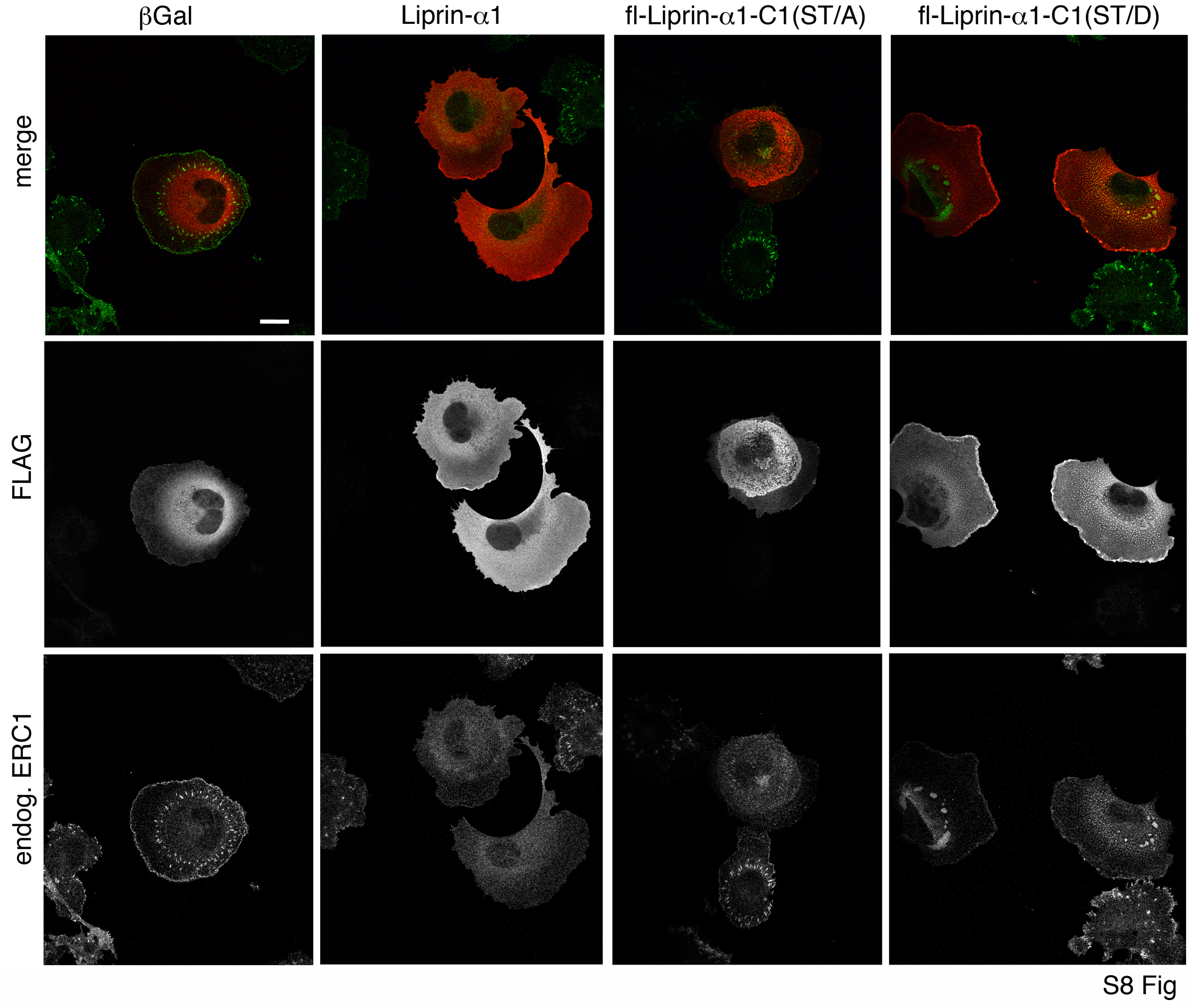

Supplement: S8 Fig — COS7 cells transfected with the indicated FLAG-tagged constructs were fixed 1 h after replating on fibronectin. FLAG (red), endogenous ERC1 (green). Bar, 20 µm. (TIF) [file pone.0337621.s008.tif]

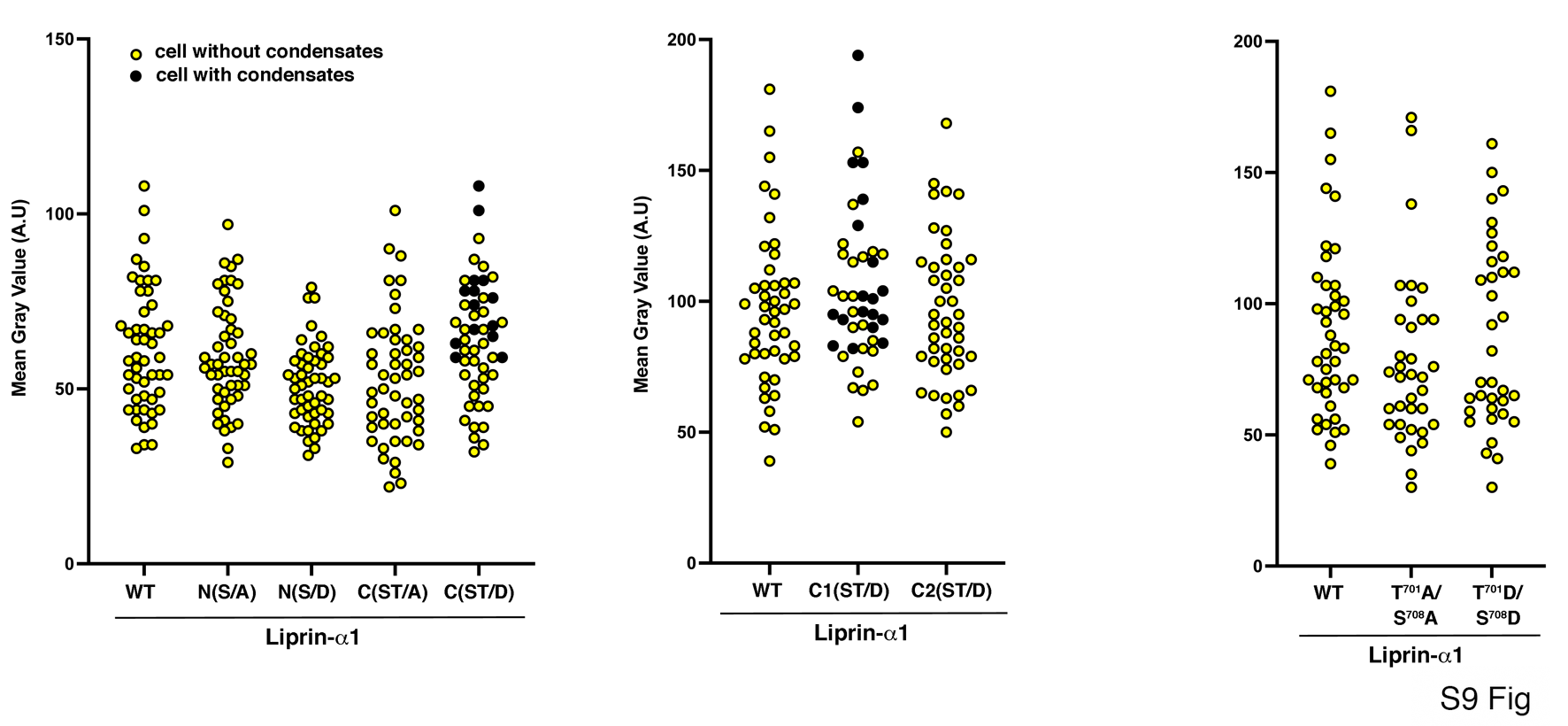

Supplement: S9 Fig — For each transfection condition, COS7 cells were analyzed after 1 h of spreading on fibronectin and processing for immunofluorescence. In each cell, the average fluorescence intensity (arbitrary units) of the FLAG-tagged Liprin-α1 construct was measured, and the presence of protein condensates was assessed. Condensates were defined as discrete round structures in the cytoplasm. Positive cells contained at least 3 small condensates or at least a large condensate-like structure. In the graph, each point represents an individual cell (n = 35–50 from two experiments): black and yellow dots indicate cells with condensates and without condensates, respectively. (TIF) [file pone.0337621.s009.tif]

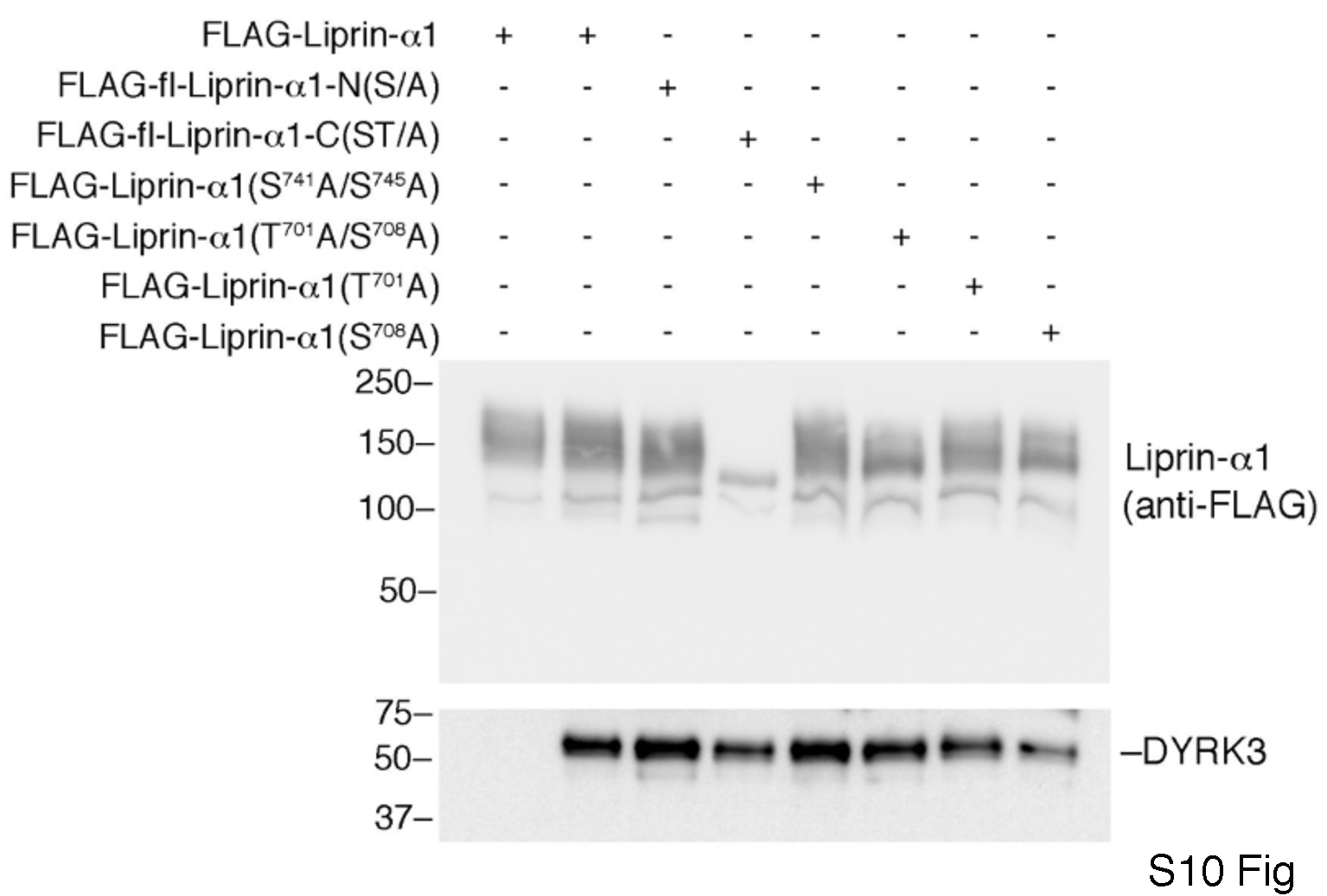

Supplement: S10 Fig — Lysates (10 µg/lane) from COS7 cells transfected for 48 h with the indicated full length Liprin-α1 constructs were analyzed by immunoblotting from Phos-Tag gels (6% acrylamide, 25 µM Phos-Tag). (TIF) [file pone.0337621.s010.tif]

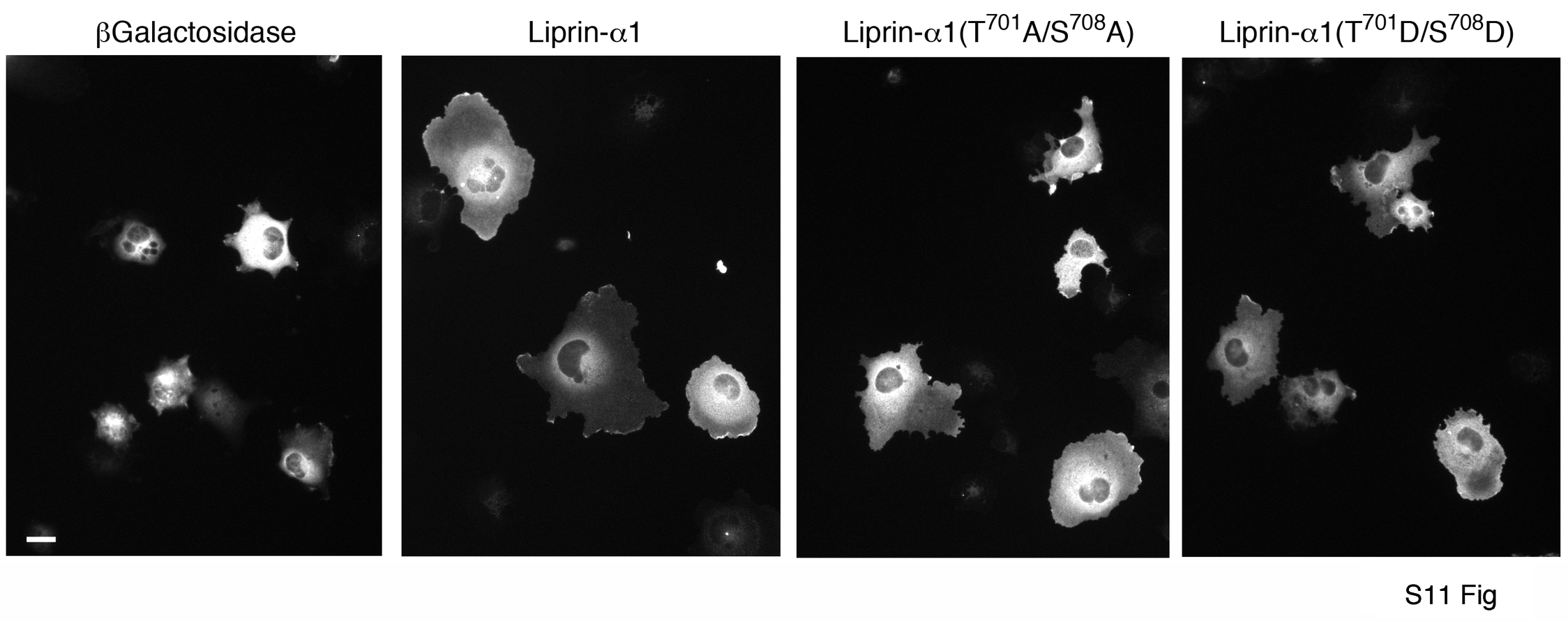

Supplement: S11 Fig — COS7 cells transfected with the indicated FLAG-tagged constructs were fixed 1 h after re-plating on fibronectin, and immuno-stained with anti-FLAG antibody. Bar, 20 µm. (TIF) [file pone.0337621.s011.tif]

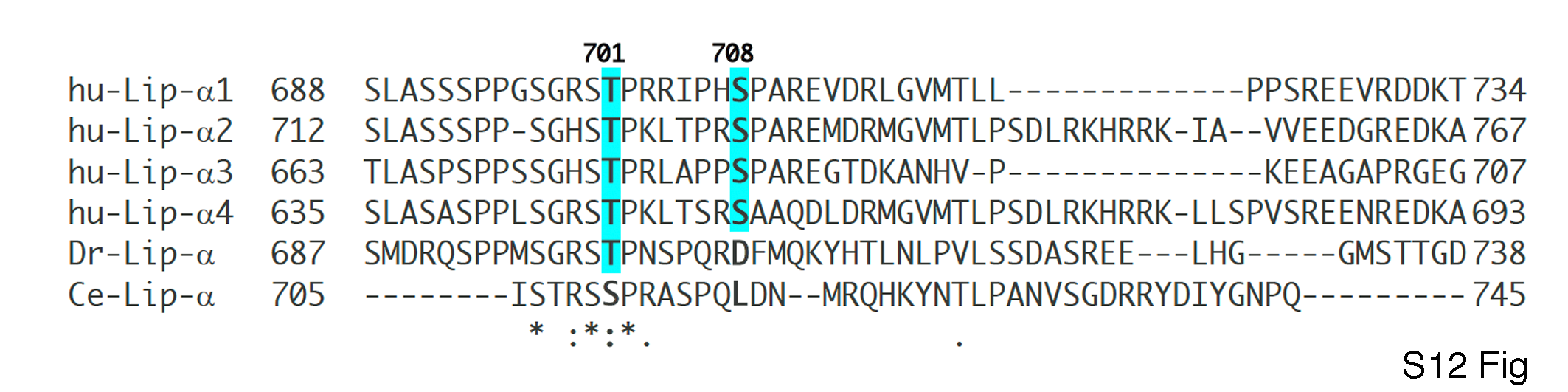

Supplement: S12 Fig — Sequence alignment of the region including residue T701 of human Liprin-α1: four human Liprin-α family members with Drosophila and C. elegans Liprin-α are shown. (TIF) [file pone.0337621.s012.tif]

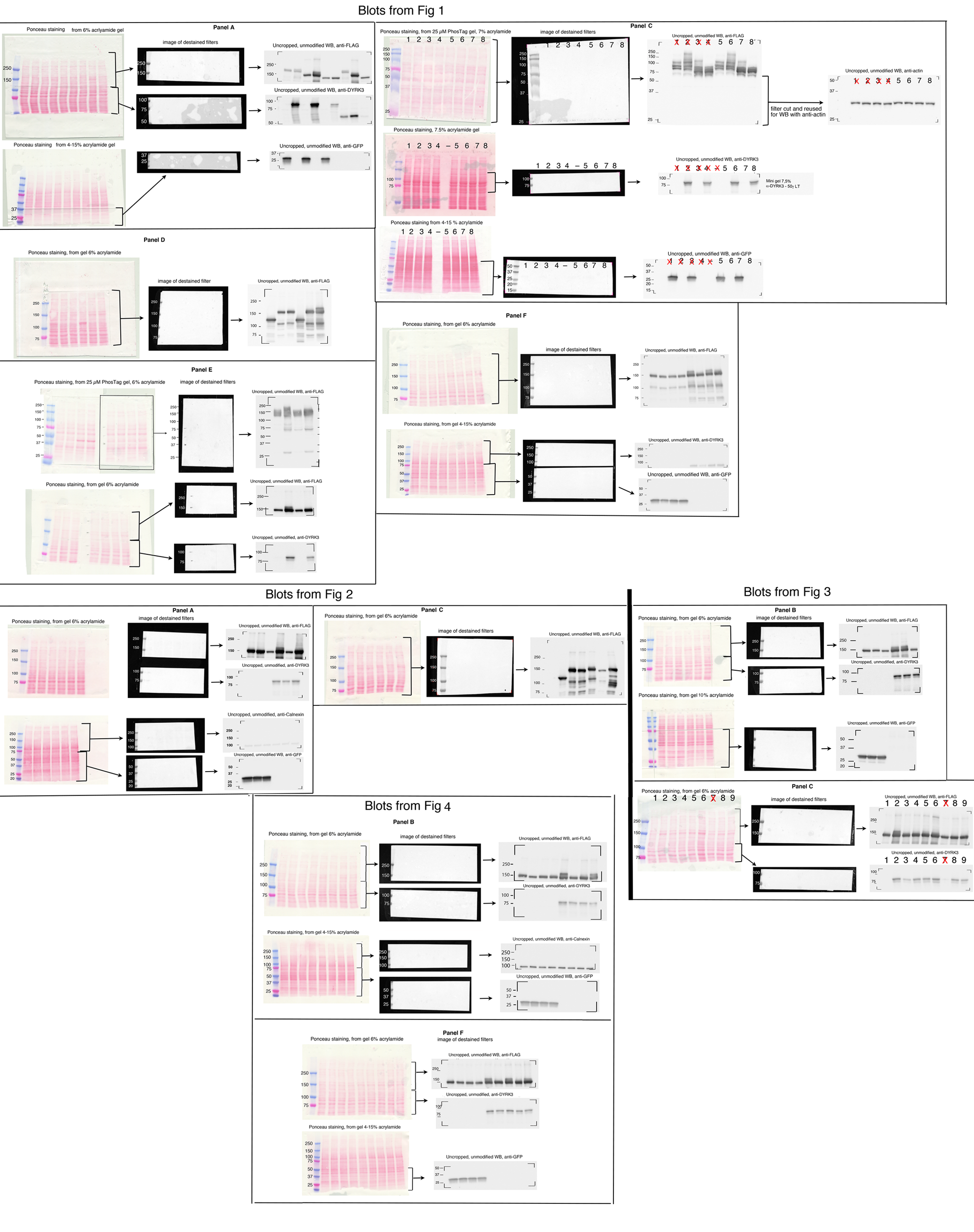

Supplement: S1 Raw images — (TIF) [file pone.0337621.s013.tif]
